# Atomic structures and oxygen dynamics of CeO_2_ grain boundaries

**DOI:** 10.1038/srep20288

**Published:** 2016-02-03

**Authors:** Bin Feng, Issei Sugiyama, Hajime Hojo, Hiromichi Ohta, Naoya Shibata, Yuichi Ikuhara

**Affiliations:** 1 Institute of Engineering Innovation, The University of Tokyo, Tokyo 113-8656, Japan; 2 Materials and Structures Laboratory, Tokyo Institute of Technology, Yokohama 226-8503, Japan; 3 Research Institute for Electronic Science, Hokkaido University, Sapporo 001-0020, Japan; 4 Nanostructures Research Laboratory, Japan Fine Ceramics Center, Nagoya 456-8587, Japan; 5 WPI advanced Institute for materials research, Tohoku University, Sendai 980-8577, Japan

## Abstract

Material performance is significantly governed by grain boundaries (GBs), a typical crystal defects inside, which often exhibit unique properties due to the structural and chemical inhomogeneity. Here, it is reported direct atomic scale evidence that oxygen vacancies formed in the GBs can modify the local surface oxygen dynamics in CeO_2_, a key material for fuel cells. The atomic structures and oxygen vacancy concentrations in individual GBs are obtained by electron microscopy and theoretical calculations at atomic scale. Meanwhile, local GB oxygen reduction reactivity is measured by electrochemical strain microscopy. By combining these techniques, it is demonstrated that the GB electrochemical activities are affected by the oxygen vacancy concentrations, which is, on the other hand, determined by the local structural distortions at the GB core region. These results provide critical understanding of GB properties down to atomic scale, and new perspectives on the development strategies of high performance electrochemical devices for solid oxide fuel cells.

Solid oxide fuel cells (SOFCs), one of the most attractive power generation technologies with high energy conversion efficiency and low emission of wasted pollutions, have been widely studied these years^1^. A critical challenge for accelerating the future commercialization of SOFCs system is to reduce the operating temperatures without degrading the material performance (especially for the electrochemical activity and carrier diffusion rate), which has been considered as a realistic approach to lower the cost and increase the durability^2^,^3^. Such demand is indeed largely dependent on the material performance, and therefore, has stimulated the field of materials science and engineering for searching novel materials with better performances^2^,^3^,^4^,^5^,^6^,^7^.

Materials in practical use are usually in the form of polycrystalline containing large density of crystal defects like grain boundaries (GBs) with abrupt structural and chemical inhomogeneity, so that it is often the case that these GBs govern their macroscopic functional properties^8^,^9^,^10^,^11^. In general, GBs were recognized to degrade material performance^11^. However, recent studies show that GBs could give rise to unique functionality which can not be realized in the perfect crystals^8^,^9^,^10^. It also holds true for the materials used in SOFC system: GBs in acceptor doped ZrO_2_ and CeO_2_ strongly degrade the oxygen diffusion kinetics^11^; while GBs on the surface of Gd doped CeO_2_ can enhance the oxygen surface reaction^9^. In this way, GBs could be either vital or fatal, and therefore, understanding and engineering these GBs would provide us a new material designing strategy in future SOFC technologies. In addition, GBs strongly affect the distribution of the dopants. Dopants are critical to the materials functionality as mentioned above, which can intrinsically determine the charge carrier type, surface reactivity and diffusion kinetics^12^,^13^. However, it is known that these dopants are usually segregated in GBs and dislocations^9^,^11^,^13^,^14^. Therefore, understanding the GB atomic structure is a prerequisite for understanding why dopants segregate in such complex crystal defects, and how they affect the resultant properties.

Despite the clear scientific importance mentioned above, however, few studies have reported on the atomistic insights into the GB structure-property relationships in fuel cell materials. This is due to the difficulties in determination of local structure and oxygen content in GBs with complex structures, as well as the local electrochemical behaviors. In this study, we show that such basic physics for GBs can be well understood at atomic scale. Pure CeO_2_ was chosen for the model materials, for its unique properties of oxygen storage capacity (OSC), that it is known to store and release oxygen vacancies associated with the valence state change of Ce from 4+ to 3+^15^. Such characteristic functionality makes CeO_2_ and CeO_2_-based materials to be one of the most promising candidate materials which are able to be involved in the whole area of an SOFC system of electrolyte^2^,^11^, anode^6^,^16^,^17^ and cathode materials^18^. Five CeO_2_ model GBs (Σ9[110]/{221}, Σ11[110]/{332}, Σ13[001]/{510}, Σ5[001]/{210}^19^ and Σ3[110]/{111}^20^ GBs were selected, where the Σ value denotes the degree of geometrical coincidence in the GB. We will denote these GBs simply as Σ3, Σ5, Σ9, Σ11 and Σ13 GB in the following text) were fabricated by bicrystal technique, in which a given type of model GB can be achieved by bonding two well defined single crystals, and allow us to precisely control the GB crystallographic orientation. Combining with the-state-of-the-art aberration-corrected scanning transmission electron microscopy (STEM) and density functional theory (DFT) calculations, local GB structure can be identified at the atomic scale, which profoundly promotes our knowledge of the structure-property correlations in materials^8^,^10^,^19^,^20^,^21^,^22^. We demonstrate that the oxygen vacancy concentration at local GB can be quantitatively evaluated by these approaches. Details for the origin of the GB dependence of oxygen vacancy content will be discussed. Moreover, the emergence of new characteristic approach of electrochemical strain microscopy (ESM), has been demonstrated for probing local oxygen reduction/evolution reactions (ORR/OER) at nanoscale in those functional oxides including CeO_2_-based materials^23^,^24^,^25^,^26^. Thus the GB electrochemical reactivity were directly identified by ESM for these model GBs. By correlating these approaches, the general physics of the CeO_2_ GBs are clarified.

## Results

### GB atomic structures and oxygen vacancy content

**Figure 1:**
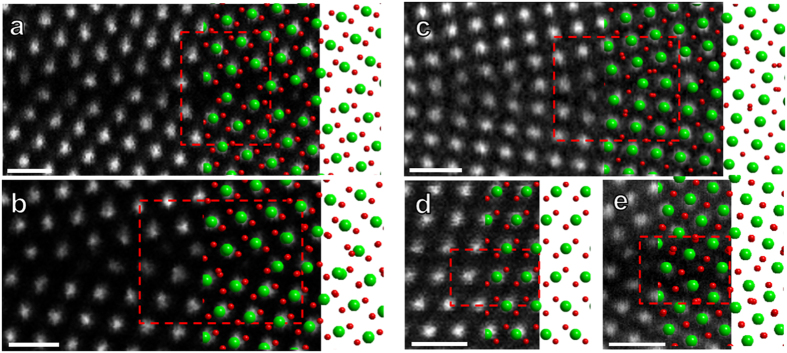
Atomic structures of model GBs obtained by HAADF STEM and theoretical calculations. (**a**–**e**) HAADF STEM images obtained from the model GBs: **(a)** Σ9 GB, **(b)** Σ11 GB, **(c)** Σ13 GB, **(d)** Σ3 GB and **(e)** Σ5 GB. The bright spots in these images correspond to the Ce atomic columns. Theoretically predicted GB models are overlaid in the right part of each STEM images. Green circle represents Ce, which shows good agreement between theoretical calculations and experimental images. It should be noted that stoichiometry GB models were shown in **(a**,**d)**, while nonstoichiometric GB models were used in **(b**,**c**,**e)**. Red rectangular region shown in each image is the unit used for EELS analysis. We took three times, twice, three times, five times and five times of the area shown from **(a**–**e)** along the GB direction, respectively. Scale bar, 0.5 nm.

Figure 1 shows typical high angle annular dark field (HAADF) STEM images of the Σ9, Σ11 and Σ13 GBs (Fig. 1a–c) obtained in this study, together with the results for Σ3 and Σ5 GBs (Fig. 1d,e) reported previously^19^,^20^. The bright spots correspond to the Ce atomic column locations. These images clearly reveal that the GB core structures are different with different GB characters (i.e., grain misorientation and interface inclination), nevertheless, all the GBs were well bonded at atomic level without amorphous nor secondary phases. The GB core structures of the Σ5 and Σ13 GB were also in agreement with those GBs observed inside polycrystalline CeO_2_ thin films^27^.

**Figure 2:**
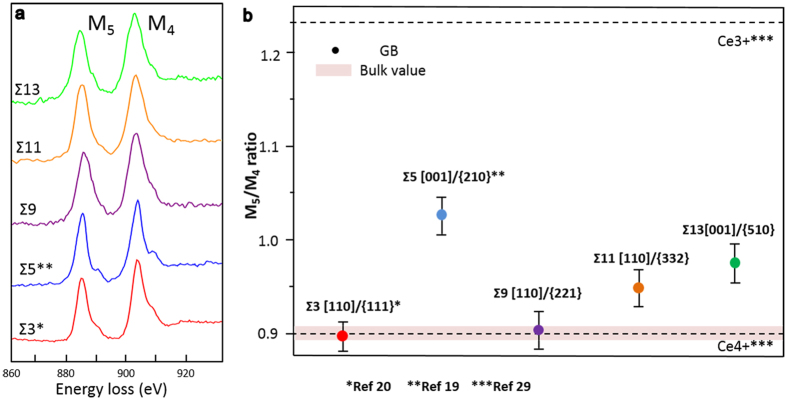
EEL spectra and their M_5_/M_4_ ratios for the model GBs. (**a**) Ce M_4,5_ edge EEL spectra obtained from the area shown in Figure 1b, (**b**) the M_5_/M_4_ ratios calculated by the positive part of second derivative of the spectrums in Figure 2a. The pink area is the bulk value taken from the bulk region of all model GBs. * from Ref 20, ** from Ref 19 and *** from Ref 29.

Subsequently we turned our attentions into the oxygen stoichiometry in GBs because it not only affects the modeling of GB structure for calculations^19^,^20^, but also be the most important factor to influence the GB physical properties. Ce M_4,5_-edge EEL spectra were taken from the area indicated by the red lines inside Fig. 1. The fine structures of the Ce M_4,5_-edges were known to be less affected by the crystal field and other bonding effects because the 4f electrons were well screened by 5s and 5p electrons^28^, which makes it difficult to identify the difference between each GBs just from the edge shapes shown in Fig. 2a (including previous results of Σ3 and Σ5 GBs^19^,^20^). However, it is suggested that the M_5_/M_4_ intensity ratios is sensitive to the chemical state of Ce, and therefore, the oxidation state of the Ce can be determined quantitatively from M_5_/M_4_ intensity ratios using the positive part of second derivative of the experimental spectra^29^. The resultant intensity ratios are shown in Fig. 2b, where 0.9 and 1.25 represent Ce^4+^ and Ce^3+^ respectively^29^. It was confirmed that Ce^4+^ is always present in the grain interior for all samples. Two essential phenomena were indicated from these results. The valence state of Ce is different with different GB characters: Ce maintains 4+ in Σ3 and Σ9 GB as those in the bulk region, however, Ce turns to be partially reduced in Σ5, Σ11 and Σ13 GBs. The formation of oxygen vacancies in CeO_2_ is always accompanied with the reduction of Ce valence state to 3+^15^, which suggests that Σ3 and Σ9 GBs maintain oxygen stoichiometry like in the bulk, while oxygen vacancies were formed in Σ5, Σ11 and Σ13 GBs. Another finding is that although Ce atoms themselves are reduced in the latter three GBs, the ‘valence state’ of Ce in each GB seems to be quite different. Since Ce usually has the discrete valence state of either 3+ or 4+, the different ‘valence state’ shown here suggests that the proportion of Ce^3+^/Ce^4+^ should be different in each GBs, and thus the oxygen vacancy concentration in each GB is expected to be different. Even though this result strongly drives us to consider the quantitative evaluation of oxygen vacancy concentration in each GB, it is difficult to determine the density of oxygen vacancies because the accurate region of GB is hard to define and the concept of such density should change according to the definition of how large the GB region is. Here the measurement unit was arbitrarily defined as illustrated in Fig. 1, of which contains characteristic structure unit of each GBs as indicated in Fig. 1a–e. By assuming that the M_5_/M_4_ ratio in the GB is composed by the linear combination of Ce^4+^(0.9) and Ce^3+^(1.25), the oxygen vacancy concentration can be evaluated^28^,^30^ (see Supporting Information for detail). This issue will be discussed later.

Then theoretical calculations were carried out to determine the atomic structure of the model GBs. Stoichiometric GB model for the three GBs were calculated first. It was found that only the most stable GB structure obtained for Σ9 GB agrees well with HAADF-STEM image shown in Fig. 1a, while the atomic structure for the Σ11 and the Σ13 predicted by the calculation show different translation state compared with HAADF-STEM images. This totally agrees with the EELS result shown in Fig. 2b that Σ9 GB is oxygen stoichiometric. Subsequently, the oxygen nonstoichiometric GB model were considered for both the Σ11 and Σ13 GB under the assumption that the oxygen vacancy formation energy in the GB is lower. The oxygen nonstoichiometric model was constructed by introducing one oxygen vacancy for the Σ11 and two oxygen vacancies for the Σ13 GB at the GB regions, of which the number of vacancies introduced was estimated from the EELS experiment. The resultant GB structures obtained from calculation are presented in Fig. 1b,c, which show perfect agreement with the experimental images respectively. It is worth mentioning that, Σ5 GB reported before displays exactly the same qualitative behavior although the oxygen vacancies were not taken into account quantitatively on purpose in the calculations^19^, which demonstrates that the present consideration is not accidental, and therefore suggests that the approaches of merging advanced STEM technique and theoretical calculations enable us to determine the GB structure even with the precise information of oxygen vacancy content. Therefore, it is concluded that the structures obtained by the theoretical calculations are plausible structures for the experimentally observed GBs. It has been known that dopants would cause the GB reconstruction and form ordered defect superstructures in ceramic GBs such as MgO and ZnO^22^,^31^,^32^. Our present results, as well as the previous study of Σ3 and Σ5 GBs shown in Fig. 1d,e, bear out that intrinsic oxygen vacancy in the GB might have virtually the same effect as those dopants, which change the local GB chemistry and lead to the GB reconstruction. These facts signify the decisive role of oxygen vacancies for the stable GB atomic structure in CeO_2_.

Much effort was spent on the quantitative analysis of oxygen (vacancy) concentration so far^14^,^33^,^34^,^35^,^36^. Kim *et al*. recently successfully developed an approach for determining the oxygen vacancy concentrations based on the information of local chemical expansion by STEM and DFT calculations^33^. Nevertheless, such method requires accurate information of lattice spacing change, which is extremely difficult for GBs due to the drastic local structural change and complicated atomic structure. For the GB, several studies successfully investigate the oxygen vacancies and dopant segregation in GBs by EELS and EDS, but only the qualitative results were available in most of the studies by far^14^,^34^,^37^. On the other hand, Jia *et al*. measured oxygen concentrations in BaTiO_3_ twin boundaries using negative Cs imaging technique by high-resolution TEM^36^, however, certain GB model for image simulations is required in advance for this technique, and the measurement would be difficult if the atomic columns are highly disordered. Therefore, the present result demonstrates that by merging atomic-scale STEM and DFT calculations, the quantitative information of oxygen vacancy concentration in atomic scale in the GB can also be precisely acquired. To further explore the difference of the degree of oxygen vacancy content in those nonstoichiometric GBs, the density of oxygen vacancies projected to each GB plane was calculated. Due to the intricate GB structure, it might be the solely proper way to make a quantitative comparison between different GBs at present stage, especially considering the fact that all the oxygen vacancies inside the GB core is distributed within a narrow space of about less than 1 nm perpendicular to the GB plane. The density is shown in Table 1 and the oxygen vacancy densities in different GBs are comparable. It is apparent that the oxygen vacancy content is 0 for the stoichiometric GB of Σ3 and Σ9 GBs, while the oxygen vacancy concentrations of nonstoichiometric Σ5 GB is higher than that of Σ13 GB, followed with the Σ11 GB.

### Physical origin of GB oxygen nonstoichiometric behavior

So far, it is revealed that the GB structure not only affect the oxygen stoichiometric condition, but also the degree of oxygen vacancy content in those nonstoichiometric GBs. We further tried to shed light on the nature of such GB-dependent oxygen nonstoichiometric behavior from the atomic structures in detail: a systematical analysis and comparison were carried out for both stoichiometric and nonstoichiometric GB structures for the entire model GBs. Here, we focused our attentions on the bonding environment of oxygen sites near the GB, because such condition is expected to be important from the view of forming oxygen vacancy. In bulk CeO_2_, a typical fluorite structure material, oxygen has fourfold coordination with Ce atoms. On the other hand, crystal defect like interfaces and GBs are always accompanied with coordination deficiencies according to previous studies^19^,^20^,^34^. In our study, the oxygen deficient sites in the GB usually take threefold coordination, with few sites have twofold coordination. As a measure of such structural distortion, we followed the evaluation method of GB oxygen vacancy densities and calculated the density of oxygen coordination deficient sites projected to each GB planes in a similar way. The results are shown in Table 1. Comparing all the densities in the stoichiometric GB model, it is apparent that the density is lower in Σ3 and Σ9 GB. The larger value of such density in the Σ11, Σ13 and Σ5 stoichiometric GBs suggests that these GB might suffer severer structural distortions. By introducing certain amount of the oxygen vacancies, the density can be largely reduced, which indicates that the structural distortion is relaxed to lower the GB energy, and as a result, oxygen nonstoichiometric GB were formed in Σ5, Σ11 and Σ13 GBs. Further investigation of correlating the structural distortions with oxygen vacancy content can be constructed now through Table 1. It clarifies that the most distorted GB of Σ5 stoichiometry GB generates the higher amount of oxygen vacancies, while the oxygen vacancy content is lower in Σ13 GB because it suffers less distortions among the two nonstoichiometric GBs, followed by the Σ11 GB and further reduction of structural distortion would lead to no oxygen vacancies for Σ9 and Σ3 GBs. There must be threshold of the density of oxygen coordination deficient sites projected to GB plane at around 14.3 nm^−2^, corresponding to a degree that GBs can tolerate certain amount of structural distortions without forming oxygen vacancies. Therefore, it is concluded that the oxygen vacancy content in CeO_2_ GBs is closely related to the degree of distortion in initial stoichiometric GB atomic structure, namely the more distorted the GB is, the more oxygen vacancies it forms.

### GB oxygen reduction reactivity

**Figure 3:**
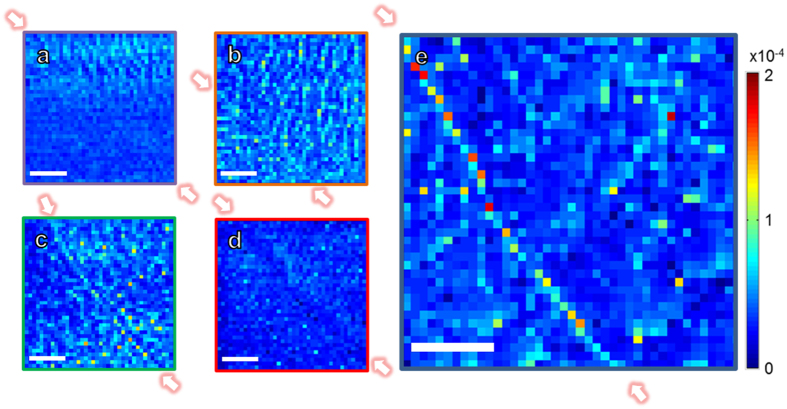
ESM mapping near model GBs. (**a**–**e**) ESM response of the model GBs of Σ9 GB (**a**), Σ11 GB (**b**), Σ13 GB (**c**), Σ3 GB (**d**) and Σ5 GB (**e**). The GB areas are indicated in between the two arrows in each figure. Scale bar, 200 nm.

The functionality of oxide materials, such as electronic, magnetic and catalytic relativities, is ultimately controlled by the oxygen vacancies distributions and dynamics^33^. The present result of GB dependent oxygen vacancy distributions consequently suggests that the corresponding local GB physical properties might be different in these GBs. Motivated by exploring the impact of the GB oxygen vacancy concentrations on the macroscopic properties, local GB oxygen electrochemical reactivity were directly measured by ESM mapping near the model GBs. In ESM, we have applied triangular voltage waveform consist of a set of pulses on a timescale of 2ms, and ESM measurements were carried out in the pulse-off state. The amplitude of triangular waveform is set to be 15 V to ensure the sample is not destructed and the measurement is reproducible. To minimize the topographic artifacts and to maximize the sensitivity, the measurements were performed in the band excitation (BE) mode^38^,^39^. By applying voltage during ESM measurements, an electric field concentrates in the tip, which also acts as electro catalytic nanoparticle (Pt in this study), resulting the oxygen vacancies to be injected or annihilated, which would induce the local strain that detectable by AFM techniques^24^. This approach is recognized as a powerful tool for detecting local oxygen electrochemical reactivity on the nanoscale^24^. The oxygen dynamics in the present study can be written in the Kroger-Vink notation as follows,


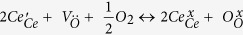


where 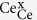 and 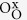 are the Ce^4+^ and O^2−^, while 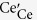 corresponds to Ce^3+^. The ESM response near each GBs are shown in the Fig. 3: the bulk part shows almost the same ESM response as in the Σ3, Σ9, Σ11 and Σ13 GBs, while an intense GB response was observed in the Σ5 GB (The corresponding ESM hysteresis loops extracted from the GB and bulk part can be found in the Supplementary Information). These results reveal that the GB reactivity is stronger than that of the bulk in the Σ5 GB, nevertheless, no obvious reactivity change is attained in the other GBs compared with that inside the bulk, demonstrating that the oxygen vacancies are critical for the surface GB reactivity. Previous studies have shown that higher GB density would lead to improved ORR kinetics at surface with similar activation energies for surface reaction, indicating that higher oxygen vacancy concentration in the GBs is likely to be the origin for oxygen incorporation enhancement^9^,^37^. Now we have provided direct evidence at nanoscale in single GBs that the oxygen vacancy concentrations greatly affect the corresponding GB oxygen reactivity, with the evidence that the Σ5 GB with the highest oxygen vacancy concentrations exhibits the strongest oxygen reactivity. Such scenario is also supported by theoretical approaches, revealing the lower energy barrier for oxygen incorporation near a GB due to the higher vacancy concentration^37^,^40^. Note that a slight intensity increase seems to appear in the Σ13 GB, but much less than the GB response in the Σ5 GB, due to the low oxygen vacancy densities in this GB. Further decrease of the oxygen vacancy densities would lead to no enhancement of GB oxygen reaction in the Σ11 GB and stoichiometric GBs of Σ3 and Σ9 GB. Although the spatial resolution of ESM is limited compared with the STEM results, the only rational possibility for the ESM change in GB should come from the GB core change. In this way, it is demonstrated that GBs can be active reaction sites for oxygen reactivity due to the oxygen nonstoichiometric GB core, which has significant implications in future optimization of material architecture in SOFC system.

## Discussions

In summary, we successfully determined the atomic structure and quantitative oxygen nonstoichiometric behavior of CeO_2_ model GBs based on the combination of atomic resolution STEM and theoretical calculations, in addition to characterizing the related GB oxygen dynamics by ESM. A simple and clear structure-property relationship in CeO_2_ GB can be concluded now: the oxygen vacancy concentrations are highly dependent on the GB atomic structures due to local structural distortions, which affects the GB oxygen reactivity. Our results highlight the paradigm of combined nanoscale characterization approaches to identify the crystallographic structure and physical properties in material interfaces, which should be applicable to a wide range of energy-related materials. Understanding such general physics of interface is significant, which enable us to predict the interface physical properties from the local interface atomic structure, and therefore, a new and effective pathway towards the rational design of functional materials for SOFC devices could be expected in future.

## Methods

### CeO_2_ thin film synthesis

The yttria-stabilized zirconia (YSZ) substrates containing Σ9, Σ11and Σ13 GBs were first fabricated by joining two YSZ single crystals at 1600 °C for 15 h in air respectively^34^. Then they were processed into substrates and the surfaces were mechanochemically polished to have a mirror finish. CeO_2_ thin films were then deposited by pulsed laser deposition (PLD) method. The substrates were initially annealed at 900 °C in the deposition chamber with an oxygen pressure of 3.0 × 10^−3^ Pa for 20 min, and then the CeO_2_ thin film was deposited. The deposition rate was set to about 3 nm min^−1^. Pure oxygen gas was introduced into the deposition chamber and then the CeO_2_ thin film was cooled down to room temperature. Out-of-plane and in-plane X-ray diffraction patterns confirmed that the CeO_2_ thin film was grown on the YSZ substrate with a cubic-on-cubic type epitaxial relationship.

### STEM observation and EELS analysis

GBs were observed using STEM (JEM-ARM200CF, JEOL Co. Ltd) operated at 200 keV. EEL spectra were acquired in STEM mode by an Enfina spectrometer (Gatan Inc). Box scan area for EELS analysis is shown in Fig. 1a–e. Multiple times of the area shown from (a–e) along the GB direction were taken, respectively. Integration time is 15s for each measurement, and box scan were carried out for five different areas in each GBs. The intensity ratio of Ce M_4,5_ edge is used for the analysis, since it is sensitive to the oxidation state of Ce and further provide quantitative information on the GB oxygen vacancies^19^,^20^,^28^,^29^,^30^.

### Theoretical calculations

For theoretical calculations, the most stable GB translation states were first determined by the static lattice calculation with GULP code for all the GBs. Buckingham potentials with potential parameters reported by Minervini *et al*. were used^41^. Both oxygen stoichiometry and nonstoichiometric model for the GBs were calculated. For nonstoichiometric GB model, oxygen atoms near the GB were systematically removed so that oxygen vacancies were introduced^19^. Then the most stable GB structure obtained including both stoichiometry and nonstoichiometric GB models were further optimized by the density functional theory (DFT) calculations using VASP code. Local-spin density approximation (LDSA) + U formalism were used and U_eff_ was set to 6 eV followed the previous studies^19^,^20^, with 2× 1× 2 k-point grids and a cut-off energy of 330 eV. It was demonstrated that GB atomic structure is little affected by the value of U_eff_^19^. All the atoms were relaxed until the residual forces were less than 0.05 eV/Å. Note that the GB structures obtained by the DFT calculation retain almost the same structures of those predicted by static lattice calculations.

### ESM measurement

AFM studies were performed with a commercial system (JEOL JSPM-5200) controlled externally by a computer via custom-written LabVIEW and MATLAB codes^42^. ESM imaging and BEPS ESM were carried out using a BE centered around the resonance frequency of the cantilever in contact with the sample, about 300~380 kHz in this case. The bipolar triangular waveform consist of 72 pulses with the maximum voltage of 15 V was applied to a Pt/Cr coated probe (BudgetSensors Multi75E-G). Backside of YSZ bicrystals are connected to the ground. Here, foreside is defined to be the probe contacting face of YSZ bicrystals. The measurements were performed with 3 V amplitude BE waveform between each pulse. Excitation generation and data acquisition was performed by National Instruments boards.

## Additional Information

**How to cite this article**: Feng, B. *et al*. Atomic structures and oxygen dynamics of CeO_2_ grain boundaries. *Sci. Rep*. **6**, 20288; doi: 10.1038/srep20288 (2016).

## Supplementary Material

Supplementary Information

## Figures and Tables

**Table 1:** Correlation between the degree of distortion and the oxygen vacancy concentrations in each model GB.

GB type	Structural distortion [nm^−2^]		Oxygen vacancy densities
	Stoichiometric	Nonstoichiometric	
Σ3[110]/{111}	8.5^a^	—	0
Σ9[110]/{221}	13.8	—	0
Σ11[110]/{332}	14.8	11.1	1.5
Σ13[001]/{510}	16.1	9.4	2.7
Σ5[001]/{210}	17.8^a^	13.9^b^	3.0^b^

The density of oxygen coordination deficient sites projected to each GB plane was calculated as a measure of structural distortion for both the stoichiometric GB and nonstoichiometric GB, while the density of oxygen vacancy projected to each GB plane obtained from EELS was shown as a measure of oxygen vacancy concentrations.

^a^data from ref. 20.

^b^data from ref. 19.
