# Supplementary material for: Atomic structures and oxygen dynamics of CeO2 grain boundaries
Source: Sci Rep. 2016 Feb 3;6:20288. doi: 10.1038/srep20288 (PMC4738319; doi:10.1038/srep20288)
Supplement: Supplementary Information [file srep20288-s1.pdf]

Supplementary Information

Atomic structures and oxygen dynamics of CeO_2 grain boundaries

Bin Feng[1], Issei Sugiyama[1], Hajime Hojo[2], Hiromichi Ohta[3], Naoya Shibata[1], and Yuichi Ikuhara[1,4,5*]

[1)] Institute of Engineering Innovation, The University of Tokyo, Tokyo 113-8656, Japan, [2)] Materials and Structures Laboratory, Tokyo Institute of Technology, Yokohama 226-8503, Japan, [3)]Research Institute for Electronic Science, Hokkaido University, Sapporo 001-0020, Japan, [4)] Nanostructure Research Laboratory, Japan Fine Ceramics Center, Nagoya 456-8587, Japan, [5)] WPI advanced Institute for materials research, Tohoku University, Sendai 980-8577, Japan

1. Evaluation of oxygen vacancy content from EELS

As introduced in the method part in the main text, EELS spectrums were taken by box scan mode. The measurement area is carefully chosen according to each characteristic GB structure, making sure to contain integral times of structure unit for each GB, as shown in Figure 1.

We will start this issue from EELS results, evaluating the concentration of Ce3+ first, then for the oxygen vacancies.

M_5/M_4 ratios were determined using the positive part of second derivative of the experimental spectra, and were determined to be 0.9 and 1.25 for Ce 3+ and Ce4+ respectively[1,2]. Ce has the discrete valence state of either 3+ or 4+, so that the whole system inside the measurement area is consist of Ce4+ and Ce3+. If we assume that the M_5/M_4 ratio is composed by the linear combination of Ce4+(0.9) and Ce3+(1.25). Thus by

$$c(\mathrm{Ce3}+) = \frac{n(\mathrm{Ce}\ 3+)}{n(\mathrm{Ce}\ 3+) + n(\mathrm{Ce}\ 4+)} = \frac{M_5/M_4 - 0.9}{1.25 - 0.9} \qquad (1)$$

where n here is the number of the atoms and c is the fraction of a given element.

the faction of c(Ce3+) inside each measurement unit shown in Figure 1b and c can be calculated[3]. Consider the case of one unit along the observation direction, then the number of Ce3+ atoms inside each measurement unit could be obtained by counting the total number of Ce atoms. If we follows the widely accepted idea from Skrodumova et al[4], that when generating one oxygen vacancy, two Ce would be reduced from 4+ to 3+. That is to say

$$2n(\mathrm{Vo}) = n(\mathrm{Ce}\ 3+) = c(\mathrm{Ce}\ 3+) * n(\mathrm{total\ of\ Ce}) \qquad (2)$$

In this way, we can acquire the number of oxygen vacancies.

2. Topography of the GBs

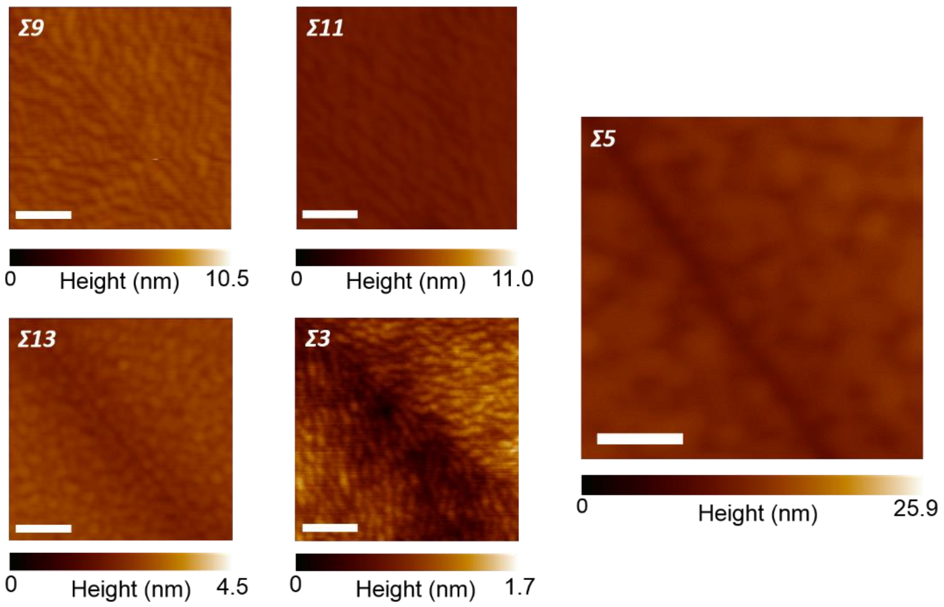

Figure S1. Topography of YSZ GBs in this study. Scale bar, 200 nm.

3. ESM hysteresis loops extracted from the GB and bulk

Figure S2 shows the ESM hysteresis loops extracted from the GB and bulk taken from the Σ5 GB. It can be seen that the ESM response in the GB is stronger than that of bulk. In addition, only the negative ESM response is different in the GB and bulk, and this can be explained as follows. Since diameter of contacting area between the probe and sample in ESM (about 10-20 nm) is much larger than the GB core width (about 1 nm), it is possible to detect the surface change when GB is uplifting regardless of the GB core width. However, it is difficult to detect any change when the GB is subsiding. Since in our AFM system, the negative response of ESM corresponds to the sample uplifting[5], we only observed difference of ESM hysteresis when applied with negative voltage.

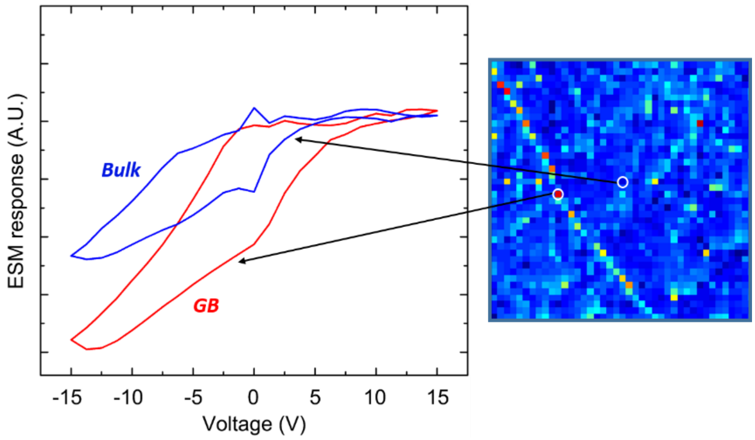

Figure S2. ESM hysteresis loops extracted from the GB and bulk taken from the Σ5 GB. The red hysteresis loop is obtained at the red point in the GB, and the blue one is obtained at the blue point in the bulk.

Reference

1 Hojo, H. *et al.* Atomic structure of a CeO2 grain boundary: the role of oxygen vacancies. *Nano Lett.* **10**, 4668-4672, (2010).

2 Fortner, J. A. & Buck, E. C. The chemistry of the light rare-earth elements as determined by electron energy loss spectroscopy. *Appl. Phys. Lett.* **68**, 3817-3819, (1996).

3 Wu, L. *et al.* Oxidation state and lattice expansion of CeO2-x nanoparticles as a function of particle size. *Phys. Rev. B.* **69**, 125415, (2004).

4 Skorodumova, N., Simak, S., Lundqvist, B., Abrikosov, I. & Johansson, B. Quantum Origin of the Oxygen Storage Capability of Ceria. *Phys. Rev. Lett.* **89**, 166601, (2002).

5 Sugiyama, I. *et al.* Spatially-resolved mapping of history-dependent coupled electrochemical and electronical behaviors of electroresistive NiO. *Sci. Rep.* **4**, 6725, (2014).
